# Rethinking dyspnea in pulmonary rehabilitation: from respiratory load and interoceptive processing to biofeedback and neurofeedback

**DOI:** 10.3389/fresc.2026.1844949

**Published:** 2026-06-24

**Authors:** Gianvito Lagravinese, Giorgio Castellana, Federico Pasqualotto, Mauro Carone, Paolo Taurisano

**Affiliations:** 1 Istituti Clinici Scientifici Maugeri IRCCS, Laboratory of Neuropsychology, Bari Institute, Bari, Italy; 2 Istituti Clinici Scientifici Maugeri IRCCS, Pneumology and Respiratory Rehabilitation Unit, Bari Institute, Bari, Italy; 3 Department of Medical and Surgical Sciences, University of Foggia, Foggia, Italy; 4 Department of Translational Biomedicine and Neuroscience, University of Bari Aldo Moro, Bari, Italy

**Keywords:** breathlessness, chronic respiratory disease, COPD, dyspnea, interoceptive processing, neurofeedback, pulmonary rehabilitation, symptom burden

## Abstract

Dyspnea in chronic obstructive pulmonary disease (COPD) is often treated as the perceptual consequence of altered airflow, lung volume, gas exchange, or work of breathing. Yet breathlessness is not a unitary symptom or a direct readout of pulmonary dysfunction. It comprises at least three partially dissociable sensations-air hunger, breathing effort, and chest tightness-with distinct physiological triggers and affective salience. Current models suggest that conscious breathlessness emerges from the interaction of respiratory motor drive, corollary discharge, sensory afferent feedback, central integration, and higher-order interoceptive inference. In COPD, this framework helps explain why dyspnea may diverge from spirometric impairment and why symptom burden can remain high despite appropriate treatment. This Perspective develops a hypothesis-generating conceptual model for pulmonary rehabilitation, rather than a systematic or scoping review. We argue that respiratory biofeedback may be relevant not only because it can modify breathing pattern, but also because it may help test whether changing perceived control, autonomic regulation, and affective responses to respiratory signals can influence rehabilitation-relevant outcomes. Our recent pilot trial in late-stage COPD is compatible with this interpretation, but it did not directly test cognitive-affective mechanisms of action. From this perspective, neurofeedback should not be considered an established treatment for dyspnea, but a plausible future translational research question for selected highly symptomatic patients. Extension of this framework beyond COPD to other chronic respiratory diseases requires condition-specific validation.

## Introduction

Pulmonary rehabilitation (PR) is a comprehensive, patient-tailored intervention that improves physical and psychological functioning in chronic respiratory disease and promotes long-term health-enhancing behaviors. Nevertheless, persistent dyspnea continues to limit functioning, emotional well-being, and engagement in rehabilitation for many patients. This mismatch suggests that pulmonary impairment may create the conditions for dyspnea without fully determining the final symptom experience (1–4).

A major problem in the literature is that dyspnea is often discussed too globally. The term is frequently used as if it referred to one homogeneous sensation, when in fact it includes at least three partially dissociable experiences: air hunger, breathing effort, and chest tightness. These sensations arise from partially distinct physiological conditions and differ in emotional salience, so grouping them too broadly can blur both mechanism and treatment interpretation (3, 5–7).

This issue is especially relevant in COPD, where dyspnea severity often correlates only weakly with physiological measures of disease severity and where patients with similar spirometric impairment may report very different symptom burden. Recent models suggest that this mismatch depends not only on respiratory mechanics, but also on how respiratory motor drive, corollary discharge, sensory afferents, and prior expectations are integrated within an interoceptive system shaped by mood, attention, and context (4, 8). In this Perspective, we use COPD as the primary clinical model and argue that PR may need to address not only the antecedents of dyspnea, but also the mechanisms through which respiratory signals become conscious suffering.

The present article is a conceptual Perspective rather than a systematic or scoping review. Accordingly, the cited literature is used to develop a plausible mechanistic framework and a hypothesis-generating research agenda, rather than to establish the balance, magnitude, or certainty of effects across all available studies. No new empirical data or statistical analyses are presented. The proposed model should therefore be interpreted as a translational framework to guide future empirical work, not as evidence that biofeedback or neurofeedback has already been clinically validated for dyspnea modulation in PR.

## Dyspnea is not unitary: distinct respiratory sensations reflect distinct mechanisms

Dyspnea comprises at least three partially dissociable sensations—air hunger, breathing effort, and chest tightness—with partially distinct physiological triggers and affective profiles. Air hunger is linked to insufficient ventilation, rising chemostimulation, and constrained lung inflation; breathing effort is more closely related to respiratory muscle activation and mechanical loading; and chest tightness is most consistently associated with bronchoconstriction. These are not merely different descriptors for the same complaint, but different forms of respiratory discomfort with different physiological and emotional characteristics (3, 5–7).

This distinction matters clinically because many studies and rehabilitation outcomes still rely on broad terms such as “shortness of breath” or “breathlessness” without specifying which component is being altered. A treatment that reduces thoracic loading may preferentially affect breathing effort, whereas an intervention that modifies anticipatory threat or interoceptive salience may have a stronger effect on the affective burden of dyspnea than on its raw sensory intensity. Broad labels may therefore obscure mechanism-specific treatment effects and fail to distinguish sensory from affective dimensions of breathlessness (5, 9).

## From respiratory drive to conscious breathlessness

In comparator models, dyspnea emerges when respiratory drive is insufficiently matched by afferent feedback, so breathlessness reflects not pathology alone but how respiratory motor command and sensory feedback are centrally processed and compared. Corollary discharge—the internal copy of the motor respiratory command—has been central to this account. In this framework, conscious breathlessness depends on the relationship between respiratory drive, sensory feedback, and the degree to which the latter satisfies or fails to satisfy the former (8, 10).

Binks’ formulation of air hunger illustrates this well. Air hunger reflects the conscious consequence of increasing inspiratory drive when ventilation cannot rise enough to satisfy it, whereas mechanoreceptive input associated with lung inflation can attenuate the sensation. Breathing effort, by contrast, is more tightly linked to the perceived activation and loading of the respiratory muscles. Different dyspnea qualities therefore arise from different relationships among motor drive, sensory afferents, and central comparison processes (5).

Recent work extends this view into a broader interoceptive framework. On this account, respiratory perception depends not only on physiological input, but also on how the brain integrates bodily signals, updates them against prior expectations, and links them to action tendencies and conscious meaning. This framework offers one plausible explanation for why patients with similar pulmonary impairment may experience different dyspnea intensity, fear, and activity avoidance (4, 8).

## Why symptom burden persists: predictive bias, affective weighting, and breathing-related brain-state modulation

Once dyspnea is framed as an interoceptive process rather than a passive sensory consequence, persistent symptom burden becomes easier to conceptualize. Recent models discuss predictive or Bayesian accounts in which priors, mood, and contextual cues influence symptom perception alongside bottom-up respiratory signaling. In that sense, dyspnea may reflect not only respiratory mechanics but also how bodily signals are interpreted and weighted within an inferential system (4, 11).

This perspective is consistent with a distinction between discriminative processes that bring respiratory sensations into awareness and cognitive-affective processes shaped by attention, expectation, mood, and interpretation. Such a distinction may help explain why dyspnea can drive inactivity, kinesiophobia, and nonadherence even when objective treatment is appropriate. However, these links should be considered plausible mechanisms requiring direct clinical testing, rather than established causal pathways in PR (12).

Electrophysiological evidence in COPD is consistent with the possibility that symptom burden involves higher-order processing of respiratory stimuli. In response to brief inspiratory occlusions, patients with COPD rated sensations as more intense and unpleasant than healthy controls and showed larger respiratory-related evoked potentials, including later P2 and P3 components. Greater P3 amplitudes were associated with higher ratings of intensity and unpleasantness, supporting the hypothesis that amplified cortical processing may contribute to dyspnea experience in some patients (13).

A further mechanistic context comes from work on breathing-brain coupling. Respiratory rhythms are linked not only to respiratory and olfactory structures but also to cortical and subcortical regions involved in salience, emotion, and cognition. In humans, respiratory phase can modulate perception, action, and higher-order cognitive processes, suggesting that breathing is not only an internal signal to be perceived but also a rhythm that helps organize neural processing (14, 15).

The clinical relevance of this mechanism for PR remains indirect. Altered breathing patterns may both reflect and help sustain brain states in which respiratory sensations become more salient and harder to regulate, whereas slower and more regular breathing may influence state regulation in ways potentially relevant to symptom tolerance, emotional reactivity, and perceived control. However, it has not yet been demonstrated that modifying breathing-related brain-state dynamics produces clinically meaningful improvements in dyspnea-related disability, adherence, or rehabilitation response.

## What does biofeedback regulate?

This model changes the meaning of biofeedback. If dyspnea is shaped by the transformation of respiratory signals into conscious distress through interoceptive, affective, and state-dependent processes, then respiratory biofeedback could be hypothesized to act at two interconnected levels. Behaviorally, it may help patients slow and regularize breathing, reduce thoracic over-reliance, and gain greater voluntary control over respiratory timing and amplitude. Experientially, it may make respiratory signals more intelligible, more controllable, and less immediately threatening.

In COPD, this suggests a testable hypothesis: biofeedback may influence not only breathing mechanics but also the psychophysiological conditions under which breathing is experienced. By increasing access to interpretable sensory evidence, it may reduce helplessness and attenuate the escalating loop in which respiratory sensations are rapidly transformed into alarm, loss of control, and withdrawal (4).

Its relevance may extend beyond breathing pattern alone. Voluntary slow breathing can influence autonomic regulation, respiratory sinus arrhythmia, heart rate variability, and broader brain-state organization. In that sense, biofeedback may alter not only how breathing is performed, but also how respiratory sensations are regulated, interpreted, and tolerated within a broader brain-body system (14, 16, 17).

The broader biofeedback literature is consistent with this interpretation, although it remains heterogeneous. In asthma, heart rate variability biofeedback has been associated with symptom improvement and reduced medication use, whereas effects on pulmonary function are less consistent. In COPD, preliminary work combining heart rate variability and pulse oximetry biofeedback has suggested benefits for function and quality of life. Taken together, these studies do not define a single mechanism of action, but they support the possibility that biofeedback may influence respiratory symptoms through autonomic, behavioral, and interpretive pathways rather than through spirometric change alone (17–19).

Our late-stage COPD pilot trial should be interpreted only as a preliminary translational observation. In hospitalized patients undergoing inpatient PR, adjunctive respiratory biofeedback did not outperform standard rehabilitation across all primary respiratory and functional endpoints, but it was associated with greater improvements in cognitive performance and depressive symptoms. This pattern is compatible with, but does not demonstrate, the hypothesis that respiratory biofeedback may influence cognitive-affective dimensions of chronic respiratory burden. The study was not designed to test mediation or mechanism of action, and it cannot establish whether any observed effects were mediated by changes in interoceptive processing, affective weighting, perceived control, autonomic regulation, or other pathways (12).

## Why neurofeedback becomes a coherent next translational question

Once biofeedback is framed as a psychophysiological adjunct rather than a purely ventilatory technique, neurofeedback becomes a plausible next research question. The rationale is not that neurofeedback treats the lung. Rather, if persistent dyspnea partly reflects dysregulated self-monitoring, maladaptive predictive weighting, affective amplification, and poor top-down regulation of respiratory salience, then more direct training of neural self-regulation may be worth exploring in selected patients.

The dyspnea-neuroscience literature already suggests that oscillatory and network-level approaches may inform rehabilitation. Neural oscillation research has been proposed as a way to clarify the time-resolved dynamics of respiratory perception, and neurofeedback has been mentioned as a possible future application, although it has not yet been established for dyspnea treatment. Mobile EEG and related approaches may also increase the feasibility of studying dyspnea in more naturalistic rehabilitation settings (20).

That does not make neurofeedback an established intervention for dyspnea. At present, it remains a hypothesis-generating translational direction. Nevertheless, if respiratory biofeedback can indirectly modulate symptom-related brain-body regulation through breathing, neurofeedback can be understood as a way of testing whether more direct training of neural self-regulation benefits those patients whose symptom burden is maintained as much by altered processing as by unresolved pulmonary impairment.

The most defensible way to introduce neurofeedback into PR research is not as a general adjunct for all patients with COPD, but as a stratified option for a subgroup with disproportionate symptom burden. These may be patients whose dyspnea is especially severe relative to spirometric or gas-exchange impairment, whose clinical picture is dominated by fear, helplessness, or activity avoidance, or whose electrophysiological profile suggests amplified cortical processing of respiratory signals. Framed in this way, neurofeedback is not a clinical recommendation, but a mechanism-oriented research question emerging from the mismatch between pulmonary impairment and experienced respiratory suffering.

## Clinical-research framework

Table 1 translates the conceptual model into clinical-research implications. It is not intended as a clinical algorithm or treatment recommendation, but as a framework for designing future rehabilitation studies capable of separating pulmonary, perceptual, affective, autonomic, and neurophysiological levels of dyspnea-related burden.

**Table 1:** Translating the conceptual model into clinical-research questions.

Model level	Clinical-research implication	Candidate measures	Current evidentiary status
Respiratory load	Continue targeting airflow limitation, altered lung volumes, ventilatory abnormalities, gas exchange, and exercise tolerance.	FEV1, SpO2, 6MWT, mMRC, exercise tolerance, symptom-limited exercise tests.	Established PR target.
Dyspnea quality	Distinguish air hunger, breathing effort, and chest tightness rather than treating dyspnea as a unitary symptom.	Multidimensional Dyspnea Profile, Dyspnea-12, symptom descriptors, sensory vs. affective dyspnea ratings.	Clinically relevant but still underused in rehabilitation trials.
Interoceptive-affective weighting	Assess fear, threat appraisal, helplessness, and avoidance as potential contributors to symptom burden.	Fear of breathlessness, activity avoidance, anxiety/depression scales, perceived control, catastrophizing-related measures.	Plausible and partially supported; direct mechanistic validation is needed.
Autonomic and breathing regulation	Test whether respiratory or HRV biofeedback improves tolerance, control, and affective responses beyond breathing pattern alone.	Respiratory rate variability, HRV, respiratory sinus arrhythmia, breathing regularity, perceived control, dyspnea unpleasantness.	Preliminary intervention evidence; mechanism remains uncertain.
Neural self-regulation	Explore neurofeedback only as a stratified research strategy for patients with disproportionate symptom burden.	EEG/ERP markers, respiratory-related evoked potentials, oscillatory measures, salience/interoceptive network markers.	Hypothesis-generating only; not an established treatment.

## Discussion

The practical implication of this Perspective is that PR should continue to treat respiratory load-including airflow limitation, altered lung volumes, ventilatory abnormalities, and gas-exchange impairment-but may also need to investigate how that load becomes conscious burden. This transformation depends on more than pulmonary dysfunction alone; it may also involve corollary discharge, afferent feedback, interoceptive integration, predictive weighting, affective salience, and breathing-related regulation of brain state (4, 8, 15).

This broader view helps explain why two patients with similar respiratory impairment may experience different degrees of fear, distress, avoidance, and disability. Peripheral mechanical and chemical signals are continuously linked to brainstem respiratory control and, from there, to wider forebrain and interoceptive networks involved in salience, emotion, expectation, and conscious perception (8, 14, 21). Recent dyspnea scholarship has described this domain as respiratory-related brain suffering, emphasizing that symptom burden depends not only on the intensity of respiratory signals but also on how those signals are filtered, anticipated, interpreted, and remembered (22).

Within this framework, respiratory biofeedback becomes relevant not only because it may change breathing behavior, but because it offers a way to test whether perceived control, autonomic regulation, and affective responses to respiratory sensations can be modified during rehabilitation. Our pilot data are compatible with that possibility, although they remain preliminary, non-mechanistic, and specific to a late-stage inpatient COPD population. Similarly, neurofeedback should not be presented as a supported treatment for dyspnea, but as a possible future translational target for carefully selected patients in whom symptom burden appears disproportionate to physiological impairment (12).

The argument advanced here has several limits. First, this Perspective relies on a narrative conceptual synthesis rather than a systematic or scoping review. This approach is appropriate for developing a mechanistic and hypothesis-generating framework, but it limits any assessment of whether the cited literature is comprehensive, balanced, or representative of the full evidence base. Future systematic or scoping reviews should more rigorously map studies linking dyspnea dimensions, interoceptive mechanisms, neurophysiological markers, and rehabilitation outcomes.

Second, much of the evidence linking interoceptive or predictive models, breathing-brain coupling, and rehabilitation outcomes remains indirect. Current evidence supports a conceptual link between breathing, interoception, arousal, attention, and affective processing, but does not yet demonstrate that modifying breathing-related brain states produces measurable improvements in dyspnea-related disability, adherence, or rehabilitation response. The proposed mechanisms should therefore be treated as clinically plausible hypotheses rather than validated therapeutic pathways.

Third, because much of the evidence discussed here comes from COPD and from experimental or theoretical work on respiratory perception, the proposed framework should be considered primarily COPD-oriented. Its possible relevance to other chronic respiratory conditions, including asthma, ILD, post-tuberculosis lung disease, long COVID, and other chronic respiratory illnesses, remains a topic for future research. These conditions may share persistent dyspnea and rehabilitation needs, but they differ in pathophysiology, symptom trajectories, inflammatory burden, ventilatory constraints, and psychological comorbidity. Therefore, the model should not be generalized across chronic respiratory diseases without condition-specific validation.

The next step is empirical. Future studies should determine which patients show the strongest mismatch between physiological impairment and subjective burden, whether electrophysiological or neuroimaging markers can identify this subgroup, and whether biofeedback- or neurofeedback-based strategies can modify outcomes relevant to rehabilitation practice. These outcomes should include not only dyspnea ratings, but also fear of breathlessness, activity avoidance, adherence, cognition, mood, and perceived control. The most useful future model may therefore be neither purely pulmonary nor purely psychological, but explicitly psychophysiological.

## Data Availability

The original contributions presented in the study are included in the article/Supplementary Material, further inquiries can be directed to the corresponding author.
